# Nutritional Challenges in Hemodialysis: Managing Protein Energy Wasting While Controlling Phosphorus

**DOI:** 10.1002/ccr3.70714

**Published:** 2025-08-01

**Authors:** Jiaxiu Deng, Weixiang Luo, Xiumei Hu, Chao Yang

**Affiliations:** ^1^ Department of Blood Purification Shenzhen People's Hospital, The Second Clinical Medical College, Jinan University, The First Affiliated Hospital, Southern University of Science and Technology Shenzhen Guangdong China; ^2^ Department of Nursing Shenzhen People's Hospital, The Second Clinical Medical College, Jinan University, The First Affiliated Hospital, Southern University of Science and Technology Shenzhen Guangdong China

**Keywords:** constipation, maintenance hemodialysis, nutritional management, protein energy wasting

## Abstract

We present a case of a 72‐year‐old male on maintenance Hemodialysis, with protein energy wasting and mild phosphoremia managed through targeted nutritional intervention that focused on high‐biological value protein intake while maintaining phosphorus control. A multimodality approach including dietary modification and supplementation led to improvement in overall nutritional status.

## 1 Introduction

Due to the influence of high metabolism, inflammation, and toxins, patients with chronic kidney disease (CKD) experience accelerated nutrient wasting and impaired absorption, resulting in protein‐energy malnutrition [1]. In 2008, the International Society of Renal Nutrition and Metabolism proposed the concept of protein‐energy wasting (PEW), which refers to a state in which CKD patients experience reduced protein and energy reserves and weight loss due to reduced nutrient intake and/or non‐specific inflammation [2]. The diagnostic criteria for PEW are shown in Table 1. PEW reduces a patient's level of physical activity and quality of life, which are independent risk factors for poor prognosis and high mortality of CKD patients [3, 4]. The incidence of maintenance hemodialysis (MHD) is 28%–54% [5, 6, 7]. PEW aggravates the poor prognosis of MHD patients and increases the risk of death. In a 1‐year follow‐up study, the all‐cause mortality rate of patients with PEW was 15.57%, which was significantly higher than that of non‐PEW patients (7.95%). Among MHD patients aged ≥ 65 years, the all‐cause mortality rate of PEW patients was 2.606 times that of non‐PEW patients [8]. PEW is often underrecognized in patients on MHD, despite it significantly impacting morbidity and mortality. Clinicians should not adopt a passive or resigned approach when managing PEW, as proactive interventions can lead to meaningful improvements in patient outcomes. This case demonstrates how a concerted multidisciplinary and multimodality approach focused on dietary optimization, involving collaboration between clinicians, dietitians, and nurses, and pharmacological intervention, and effective dialysis can be impactful.

**TABLE 1:** Readily utilizable criteria for the clinical diagnosis of PEW in CKD [2].

Criteria
Serum chemistry
Serum albumin < 38 g per 100 mL
Serum prealbumin (transthyretin) < 30 mg per 100 mL (for MHD patients only; levels may vary according to GFR level for patients with CKD stages 2–5)
Serum cholesterol < 100 mg per 100 mL
Body mass
BMI < 23
Unintentional weight loss over time: 5% over 3 months or 10% over 6 months
Total body fat percentage < 10%
Muscle mass
Muscle wasting: reduced muscle mass 5% over 3 months or 10% over 6 months
Reduced mid‐arm muscle circumference area (reduction > 10% in relation to 50th percentile of reference population)
Creatinine appearanced
Dietary intake
Unintentional low DPI < 0.80 g kg^−1^day^−1^ for at least 2 months for dialysis patients or < 0.6 g kg^−1^day^−1^ for patients with CKD stages 2–5
Unintentional low DEI < 25 kcal kg^−1^day^−1^ for at least 2 months

*Note:* At least three out of the four listed categories (and at least one test in each of the selected category) must be satisfied for the diagnosis of kidney disease‐related PEW.

Abbreviations: BMI, Body Mass Index; DEI, dietary energy intake; DPI, dietary protein intake; GFR, glomerular filtration rate.

## 2 Case Presentation

This case involved a 72‐year‐old male MHD patient (patient information in Table 2). Doctors prescribed dialysis (Table 3), with the first hemodialysis performed on November 20, 2018. On July 13, 2023, the biochemical markers of the patient were determined (Table 4). Abdominal computed tomography examination and other auxiliary examinations were performed.

**TABLE 2:** Patient information.

Items	Contents
Basic information	Married; retired; dry weight: 69 kg; height: 1.75 m
Past medical history	(1) The patient underwent a colonoscopy in 2011, and two hyperplastic polyps were found and removed.
	(2) In 2018, the patient was admitted to the hospital since his serum creatinine was too high for the last 17 years; arteriovenous fistula (AVF) plasty of the left upper extremity was performed.
Present medical history	(1) Regular hemodialysis three times a week
	(2) Blood pressure: 80–130/40–80 mmHg; heart rate: 57–69 beats/min during dialysis.
	(3) Symptoms: insomnia, decreased activity and diet Constipation
Clinical diagnosis	Chronic kidney disease stage 5; renal anemia; coronary atherosclerosis; polycystic kidney; cardiac insufficiency

**TABLE 3:** Dialysis prescription.

Items	Contents
Vascular access	AVF
Dialysis mode	Hemodialysis twice a week. Hemodiafiltration once a week.
Ultrafiltration volume	1.2–1.5 L
Dialyzer	High flux
Dialysate flow rate	500 mL/min
Dialysate	Calcium: 1.5 mmol/L; potassium: 2.0 mmol/L; sodium: 140 mmol/L; sodium bicarbonate
Blood flow rate	250 mL/min
Anticoagulant	Low molecular weight heparin: 2000 units

**TABLE 4:** Biochemical indexes before and after nursing.

Indicators	2023‐7‐13	2023‐9‐01	Criterion	% change
Hemoglobin	930	1080	≥ 1100 g/dL	+16.0%
Serum albumin	339	386	350–550 g/dL	+13.8%
Potassium	4.27	4.97	3.5–5.5 mmol/l	+16.4%
Calcium	2.06	2.16	2.10–2.50 mmol/L	+4.9%
Phosphorus	1.52	1.67	0.87–1.45 mmol/L	+9.7%
Serum creatinine	1100	1179	44–133 mmol/L	+7.2%
Serum iron	6.63	10.09	9.0–30 μmol/L	+52.2%
Transferrin saturation	15.82	25.81	20%–50%	+63.1%

On July 13, the dietitian involved the patient and his family members in jointly recalling in‐person the types and amounts of food consumed over the past 3 days. The patient's dietary structure was assessed and reflected (Table 5). Dietary evaluation was conducted by a dietitian, and the intake of proteins and calories was calculated. The intake of protein was 55 g, and that of calories, 1297.1 kcal. The patient's protein intake was 55 g/day, falling 28 g/day (34%) below the recommended 1.2 g/kg/day, and the patient also suffers from a lack of energy, falling 772.9 kcal/day (37%) below the recommended 30 kcal/kg/day for hemodialysis patients, both contributing to ongoing protein‐energy wasting. According to the diagnostic criteria for PEW [2], and pertinent to this case study, the patient had a serum albumin level < 380 g/dL, BMI < 23 kg/m^2^, weight loss of 2 kg, unintentional low DPI < 0.8 g/kg, and unintentional low DEI < 25 kcal kg‐1 day‐1 in the previous 2 months.

**TABLE 5:** Comparison of dietary intake.

Item	Recommended	Pre‐intervention	Actual
Serum albumin	380 g/dL	339 g/dL	386 g/dL
BMI	≥ 23 kg/m^2^	22.5 kg/m^2^	23.2 kg/m^2^
Dietary intake	Breakfast: 50 g of Chinese steamed buns(or 240 mL of thick white porridge), egg whites 50 g (or milk 250 ml); Lunch: rice 150 g, 200 g of meat (50 g of pork, 100 g of fish, and 50 g of beef), vegetables 250 g; Chayote squash 100 g, fruits 100 g; Dinner: rice 150 g, chicken 100 g, vegetables 250 g; Chayote squash 100 g; fruits 100 g.	Breakfast:Chinese steamed buns with meat filling 60 g; milk 100 ml; Lunch: rice 150 g, pork 50 g, beans 60 g, freshwater perch 80 g, Chinese cabbage 100 g; Dinner: rice 120 g, pork 50 g, lettuce 120 g.	Breakfast: rice noodles 150 g, egg whites 50 g; Supplemental meal: lotus root powder pudding 100 g; Lunch: rice 150 g, sweet potatoes 100 g, pork 100 g, freshwater perch 70 g, lettuce 250 g, winter melon 100 g, apples 50 g and strawberries 50 g; Supplemental meal: lotus root powder pudding 100 g; Dinner: rice 120 g, sweet potatoes 100 g, chicken100g, Chinese flowering cabbage 250 g, winter melon 100 g, apples 50 g and pears 50 g; olive oil: 30 mL.
Dietary protein intake(DPI)	69 kg × 1.2 g/kg = 82.8 g; high biological value protein was more than 50% (42 g)	Total protein: 55 g; high biological value protein: 40.4 g	Total protein: 83.2 g; high biological value protein: 58.6 g
Dietary energy intake(DEI)	69 kg × 30 kcal = 2070 kcal	1297.1 kcal	2141.9 kcal

## 3 Treatment Plan

(1) The patient was advised to increase egg white intake from two to three egg whites, take a compound alpha keto acid preparation, and increase oral supplementation [9]. (2) Erythropoietin, 10,000 units was injected subcutaneously once every 2 weeks and later adjusted to once every 10 days. (3) Oral iron supplements were initiated, one tablet daily, and later adjusted to two tablets once a day. (4) Oral phosphate binders were to be maintained at three times a day with meals. (5) The intake of sodium bicarbonate was maintained at two tablets three times a day; folic acid tablets 5 mg, three times a day; midodrine, plavix, lipitor, and isosorbide mononitrate (one tablet each daily); diazepam (two tablets before bedtime).

On September 1, 2023, the patient's dry weight was 71 kg and blood tests were performed for hemoglobin, serum iron, and serum albumin levels (Table 4).

## 4 Dialysis‐Related Hypotension Care

The patient had complications of PEW, low serum albumin, and a low systolic blood pressure that frequently decreased to < 90 mmHg during dialysis. This necessitated an adjustment to the hemodialysis nursing program for the patient. First, the temperature of the dialysate was set at 35.5°C to prevent hypotension during dialysis, and the patient was kept warm by using quilts. Second, the patient was encouraged to eat some food, for example an egg, when the hemodynamics were stable [9]. Third, 100 mL of 50% glucose was administered by continuous infusion. Fourth, drugs to enhance the sympathetic response of the body were administered orally. Fifth, when the patient had low blood pressure, ultrafiltration was stopped. Sixth, to prevent a sudden change of position and postural hypotension, the patient was instructed to sit up for 30 s before getting off the bed and stand for 30 s before moving further. Seventh, we instructed the patient to use erythropoietin as prescribed to improve the hemoglobin level.

### 4.1 Dietary Care

#### 4.1.1 Recommended Intake

We instructed the patient to choose foods with a phosphorus/protein ratio < 12 mg/g, for example, egg white, tilapia, chicken, beef, and lean pork [10, 11]. According to the standard weight of the patient, protein intake was calculated as 83 g based on the standard of 1.2 g/kg per day, and the intake of high biological value protein was more than 50% (42 g) to maintain the daily requirements and increase the serum albumin level [12]. The daily intake of calories was 2070 kcal based on the standard of 30 kcal/kg. The standard daily protein intake was allocated as follows: 49 g of high biological value protein contained in 350 g of egg, meat, and milk, which can generate 630 kcal; the other 34 g of protein came from non‐high biological value protein: 24 g of protein came from 300 g of cereal and sweet potato, which generated 1080 kcal, and the remaining 10 g of protein came from 500 g of vegetables and 70 g of Chinese steamed buns with meat filling, which generated 150 kcal. The total amount of calories was 1835 kcal.

Dietary intake information in Table 5. The protein and energy in every 100 g of food in Table 6.

**TABLE 6:** The protein and energy in every 100 g of food.

Food	Protein (g)	Energy (kcal)	Food	Protein (g)	Energy (kcal)
Meat Bun	7.3	230	Freshwater perch	18.6	105
Rice	2.6	116	Flowering cabbage	2.8	25
Porke	20.3	143	Lettuce	1.3	15
Bean	2.5	30	Egg whites	11.6	60
Winter melon	0.4	11	Chicken	19.3	167
Apple	0.2	52	Pears	0.4	44
Straw berry	1	30	Sweet potatoes	1.1	99
Rice Noodles	0.9	109	Olive oil	0	899
Lotus root powder pudding	0.2	370			

#### 4.1.2 Dietary Intake Instruction(High‐Protein, High‐Calorie, Low‐Phosphorus Intake)

Breakfast: 70 g of Chinese steamed buns (or rice noodles 150 g), egg whites (50 g). Lunch and dinner: 300 g of meat (chicken, beef, lean pork, fish, etc.), 500 g of vegetables, rice, and grains (sweet potato). We instructed the patient to take 200 g of meat (50 g of pork, 100 g of fish, and 50 g of beef). For dinner, 100 g of pork and fish were selected. The pork, fish, and beef were not to be fried. The dietitian monitored dietary intake: instruct the patient's family members to use a precise household food scale to weigh and photograph food items, ensuring that raw and cooked foods are weighed separately. The dietitian had patients or their family members weigh food, take photos, and keep a diet diary. We monitored patients' dietary compliance by WeChat, phone, and in‐person.

#### 4.1.3 Nutritional Supplements

We instructed the patient to take protein‐based oral nutritional supplements with low phosphorus, low potassium, and high energy density for kidney disease [11] to improve the patient's serum albumin and pre‐albumin levels and increase the mid‐arm muscle circumference, as an increased intake of proteins promotes and maintains muscle growth [13]. The patient took oral nutritional supplements as recommended by the dietitians and doctors.

#### 4.1.4 Constipation Care(Increase Dietary Fiber)

In addition to PEW, the patient experienced constipation. The patient was instructed to increase dietary fiber intake; for example, the consumption of celery, leek, and Chinese cabbage. A variety of vegetables was consumed, up to 750 g every day [14]. The vegetables were not cut to increase the intestinal cellulose capacity and facilitate defecation. In addition, we instructed the patient to eat cucumber, apple, kiwi fruit, and other sources of dietary fiber and vitamins. On July 22, the patient's constipation was relieved.

## 5 Conclusion and Results

The patient in this case is an elderly male with significant protein‐energy wasting. The precision nutrition intervention prioritized the patient's protein and energy intake, improving his nutritional status. See Table 3 for details. The nutritional calculation method provides a reference for the nutritional guidance of other patients. For patients with poor appetite, priority is given to meeting their dietary intake.

## 6 Discussion

### 6.1 The Higher Risk of Protein‐Energy Wasting Than Mild Hypophosphatemia

On the premise of ensuring protein intake, the patient limited the intake of inorganic phosphorus, such as beverages, flavorings, and processed foods, and took phosphorus binders with regular meals as directed. Because the PEW of hemodialysis patients has greater impact on prognosis than hyperphosphatemia, hemodialysis patients with PEW should first correct malnutrition rather than strictly control hyperphosphatemia [15, 16]. Restricting phosphorus intake by limiting high‐quality protein is associated with higher mortality [17]. Most meat products are high in phosphorus. Therefore, many patients restrict their intake of meat due to concerns about excessive phosphorus intake, which leads to insufficient protein intake. In this case, we selected high‐quality, high‐protein foods for the patient. Phosphorus primarily exists in animal‐based and plant‐based foods in the form of phosphates, which have a certain degree of solubility in water. When food is placed in boiling water, some of the phosphates dissolve into the water, achieving the goal of removing some phosphorus. This ensured adequate protein intake while limiting organic phosphorus. We also instructed the patient to set an alarm clock to remind himself to take phosphorus‐lowering medication during meals, which prevented hyperphosphatemia. However, for other patients with high blood phosphorus and high creatinine levels, it is important to guide them in controlling excessive meat intake.

### 6.2 The Importance of Resolving Constipation

When patients suffer from constipation, it can increase the absorption of phosphorus in the intestines and reduce the excretion of phosphorus bound by phosphate binders. Therefore, patients increase dietary fiber intake to promote bowel movements, reducing the reabsorption of phosphorus, and increasing the patient's appetite.

### 6.3 Increase Protein Intake While Controlling Blood Urea Nitrogen

We precisely calculated the daily protein intake according to the guidelines to avoid excessive protein consumption and prioritize high‐biological‐value protein sources, which contain essential amino acids required by the human body and produce relatively fewer metabolic waste products [18]. At the same time, we distributed the protein intake across multiple meals to avoid large single intakes, thereby reducing the generation of blood urea nitrogen [19]. We also ensure the intake of an appropriate amount of carbohydrates to reduce the catabolism of protein, which in turn lowers the production of blood urea nitrogen [20].

## 7 Limitation

As the patient's C‐reactive protein (CRP), tumor necrosis factor‐α (TNF‐α) and Interleukin‐6 (IL‐6) levels were not tested, the malnutrition inflammation score (MIS) was not used to assess their nutritional status. In this case, we lacked the assessments for sarcopenia or muscle strength, such as grip tests, sit‐to‐stand test, and mid‐arm circumference. The generalizability of this case is limited by the complexity of the precision nutrition intervention, which can only be applied to patients who urgently need to improve their nutritional status. Moreover, it requires significant time and effort to monitor patients' dietary compliance. Patients need to adhere to proper nutrition over the long term.

## Author Contributions

**Jiaxiu Deng:** methodology, project administration, supervision, writing – original draft. **Weixiang Luo:** writing – review and editing. **Xiumei Hu:** resources. **Chao Yang:** methodology.

## Disclosure

The authors have nothing to report.

## Consent

A written informed consent was obtained from the patient to publish this report in accordance with the journal's patient consent policy.

## Data Availability

The data that support the findings of this study are openly available.
